# Social support, social networks, and mental health of six refugee subgroups in Arizona: Findings from a pilot study

**DOI:** 10.1371/journal.pmen.0000553

**Published:** 2026-02-19

**Authors:** Mee Young Um, Youn Kyoung Kim, Arati Maleku, Zoe Baccam, Sabaa Abdullah, Jamil Shahin, Aiman Hesswany, Tom Taknan, Muktar Sheikh, Pitchou Mulongo

**Affiliations:** 1 School of Social Work, Watts College of Public Service and Community Solutions, Arizona State University, Phoenix, Arizona, United States of America; 2 School of Social Work, Louisiana State University, Baton Rouge, Louisiana, United States of America; 3 College of Social Work, Ohio State University, Columbus, Ohio, United States of America; 4 Arizona Allnation Resource Refugee Center, Phoenix, Arizona, United States of America; 5 Syrian Community Service Center, Phoenix, Arizona, United States of America; 6 Chin Burmese Community in Arizona, Phoenix, Arizona, United States of America; 7 Somali Association of Arizona, Phoenix, Arizona, United States of America; 8 DRC Community of Arizona, Phoenix, Arizona, United States of America; PLOS: Public Library of Science, UNITED KINGDOM OF GREAT BRITAIN AND NORTHERN IRELAND

## Abstract

Mental health care is vital to refugee integration, yet current frameworks often lack the cultural sensitivity needed to address distinct subgroup needs. This limits the understanding of how cultural and experiential factors shape refugees’ mental health and responses to care. Research that predominantly treats refugees as a homogeneous group overlooks these differences, hindering the development of effective, nuanced interventions. Although social connectedness is widely recognized as protective for mental health, prior research often has simplified this to general social support measures, overlooking the unique structure and function of refugees’ social networks. To address these gaps, this pilot study used a community-based participatory approach and egocentric network data to examine social support, social network characteristics, and mental health across six refugee groups—Bhutanese, Burmese, Congolese, Iraqi, Somali, and Syrian—resettled in Arizona, a key U.S. resettlement location. Our sample (*N* = 150) was 53.0% male and 47.0% female with an average age of 41.4 years; 31.3% reported no formal education and 44.7% were unemployed. Univariate analyses revealed significant differences in sociodemographic characteristics, social support levels, social network characteristics, and mental health statuses (psychological distress and suicidal thoughts) among refugee subgroups. These findings underscore the need for culturally responsive interventions that encourage cross-cultural engagement, expand community support, and foster diverse networks to support refugee mental health and integration. This study is the first to employ egocentric network data to compare social networks and mental health indicators among Arizona’s refugee subgroups.

## Introduction

Refugee resettlement is an inherently complex and often traumatic process, shaped by a combination of challenging premigration experiences and significant socioeconomic and cultural hurdles in the host country. One of the most critical yet often underaddressed dimensions of refugee resettlement is mental health. Having endured violent and traumatic premigration experiences, many refugees face heightened risk of adverse mental health outcomes such as depression, anxiety, posttraumatic stress disorder (PTSD), suicidal ideation, and psychological distress [1–3]. These challenges can intensify during resettlement, as refugees navigate socioeconomic stressors such as unemployment, language barriers, social isolation, and systemic inequities in healthcare access [4]. Although mental health care is crucial for successful refugee integration, current frameworks often lack the cultural competence needed for effective interventions, resulting in an insufficient understanding of how best to address refugees’ distinct needs [4]. Addressing this gap is critical, because effective treatment can mitigate the long-term impacts of trauma from premigration, migration, and resettlement experiences.

Traditional mental health interventions, including trauma-focused therapies, are central to addressing mental health challenges but often overlook the complex cultural backgrounds of refugees [5–7]. Particularly, the prevalent one-size-fits-all approach in Western mental health models often fails to meet the distinct needs of refugee subgroups, overlooking cultural and experiential differences that influence both mental health and responses to care [5–8]. Recognizing these limitations, scholars have advocated for a refugee-informed approach that addresses the unique traumas of migration, acknowledges human rights violations, and considers specific forms of trauma, such as humiliation [9]. Moreover, the tendency in research to treat refugees as a homogeneous group further obscures these critical differences, impeding the development of interventions that effectively meet the needs of diverse refugee subgroups [10,11]. A more nuanced approach that recognizes cultural, linguistic, and experiential diversity is essential, shifting away from generalized models and toward tailored care to support effectively the unique and intersectional experiences that shape refugee mental well-being [12]. Disaggregating data across these sociodemographic profiles is essential to define the problem’s scope, reveal differences among subpopulations, and increase the visibility of vulnerable groups [13].

A large body of research has documented the crucial role of social connectedness in mitigating mental health challenges among refugees [2,5,7,14,15]. Social support networks are thus essential for not only understanding the prevalence of mental health issues but also identifying effective interventions to improve well-being. Social networks among refugees vary widely, shaped by distinct cultural backgrounds, language barriers, and past trauma [16]. These factors influence whether refugees rely mainly on coethnic networks or also locals, which can enhance resilience and adaptation. However, prior research is limited because social connectedness is often assessed by a general measure of social support without accounting for the structure and function of refugees’ social networks [17]. Whereas social support reflects the functional aspects of relationships, social networks encompass both structural and functional dimensions, offering a fuller understanding of how these relationships influence mental health [18,19]. Moreover, most research on refugee social networks has been qualitative, with comparatively fewer quantitative studies [20].

To address these gaps, this pilot study used egocentric (personal) network data to explore social support, social network characteristics, and mental health among six refugee groups resettled in Arizona, a major U.S. resettlement site. Egocentric network data offer insights into participants’ social connections (i.e., alters) and the interrelationships of these connections, providing a closer look at how these networks are associated with mental health outcomes [18]. This approach enables the development of more culturally relevant interventions that address the complexity of refugees’ social environments. Further, this study challenges the common practice of treating refugee populations as homogeneous in research and policy, instead offering a nuanced perspective on how variations in social networks and cultural contexts may relate to mental health across refugee groups. The findings can inform culturally sensitive interventions, enhance service provision, and guide policy to better support the integration and well-being of refugee subgroups in Arizona.

### Local context

Arizona ranked fifth in the United States for refugee resettlement in 2023 [21] and offers a unique setting to examine refugee social networks and mental health. The Arizona Refugee Resettlement Program, led by the Department of Economic Security, plays a crucial role in helping refugees adapt to life in the state. It provides short-term services such as housing, cultural orientation, case management, and referrals for healthcare and employment, typically within the first 90–180 days of arrival [22]. As of July 2024, Arizona had accepted 4.2% of all refugees entering the country that year [23], highlighting its importance in national resettlement efforts. However, Arizona ranks 49th nationwide in overall mental health according to the State of Mental Health in America report, which evaluates both adult and youth populations [24]. This ranking reflects several key indicators, including a high percentage of adults reporting serious thoughts of suicide, a large proportion of individuals with mental illness who report unmet treatment needs, limited availability of mental health providers, as well as broader issues related to insurance coverage and access to treatment [24]. Thus, while the state’s infrastructure supports refugees’ initial integration, long-term access to services—particularly mental health care—remains limited. This places added responsibility on local community organizations and highlights ongoing challenges in integrating comprehensive mental health services into resettlement programs. Arizona lacks targeted mental health programs that address the specific culturally embedded needs of refugees [25]. Many refugees must navigate mainstream mental health services that often do not accommodate the unique traumas they have experienced, creating significant gaps in care [26]. One major barrier to timely care is the application process for the Arizona Health Care Cost Containment System (AHCCCS), which is the Arizona Medicaid program that intends to provide healthcare coverage to low-income individuals including many refugees [27], can take more than 45 days, posing potentially life-threatening delays for those in urgent need of psychological support. This bureaucratic hurdle compounds the challenges refugees face, including trauma, language barriers, and socioeconomic stressors, underscoring the need for more responsive mental health resources.

Although refugee mental health research has occurred in Arizona, it remains limited, especially for the state’s diverse refugee subgroups. For instance, studies have shown that half of refugee women who screened positive for mental health disorders at an Arizona health clinic did not pursue further treatment, indicating substantial gaps in culturally responsive care [28]. These findings highlight the critical need for localized, research-informed interventions that address the social, cultural, and emotional complexities of refugee mental health in Arizona.

### Mental health of diverse refugee groups

Psychological distress and suicidal ideation are major concerns among refugee populations, with rates significantly higher than those of nonrefugees in many contexts [2,14,29,30]. Recent studies indicated that up to 33% of resettled refugees experience psychological distress and 20.5% report suicidal ideation [1,31]. A meta-review of systematic reviews on suicide and suicidal behavior among refugees highlighted the elevated rates of these mental health challenges compared to native populations and noted the limited empirical evidence on specific refugee groups [14].

Levels of psychological distress and contributing factors vary significantly across refugee groups. For instance, Bhutanese refugees in the U.S. Midwest reported psychological distress (18.7%), PTSD (8.1%), and suicidal ideation (7.7%), with predictors of poor mental health including social isolation, inadequate mental health support, and poor physical health [32,33]. Similarly, a study on Burmese refugees in four U.S. states found that 34.3% experienced psychological distress, with factors such as low English proficiency, postmigration difficulties, including challenges in securing stable housing, employment barriers, cultural adjustment stress, discrimination, and navigating complex healthcare and legal systems, and concerns for family members abroad contributing to their mental health challenges and social support serving as a protective factor [15,34,35].

Among Congolese refugees in Rwandan refugee camps, poor mental health and a lack of social connectedness were strongly linked to suicidal ideation [5]. For Congolese refugees in Kenya, 52.8% reported psychological distress, often worsened by barriers like limited access to healthcare and a lack of community roles; however, those who sought religious support experienced a reduction in distress [36]. Among Iraqi refugees in the United States, approximately 50.0% reported anxiety, depression, and emotional distress, with 31.0% identified as at risk of PTSD [37]. Premigration trauma emerged as a major risk factor, whereas resilience played a protective role in mitigating mental health challenges [38].

Among Somali refugees in Kenya, prior research found that premigration trauma exposure, lack of education, and female gender were associated with increased mental health issues, including PTSD, depression, and anxiety [39]. In contrast, a willingness to share problems with social networks was linked to reduced mental health issues [39]. Similarly, Syrian refugees experience alarmingly high rates of mental health challenges. A meta-analysis indicated that 33% of Syrian refugees in high-income Western countries suffer from at least one common mental disorder, such as anxiety (40.0%), depression (31.0%), or PTSD (31.0%) [40]. Another review reported similar rates for PTSD (43.0%), depression (40.9%), and anxiety (26.6%) among Syrian refugees, with traumatic experiences, gender, and postmigration conditions playing significant roles in shaping mental health outcomes [41,42]. These findings underscore the considerable variability in mental health outcomes across refugee groups, highlighting the importance of tailored interventions that address both shared and unique experiences in these populations.

### Role of social support and social networks

Social isolation is a well-established predictor of mental health issues among refugees [43]. Forced migration disrupts refugees’ social networks, separating them from vital support systems in their home countries and often leaving them to navigate resettlement without close family members [2,14]. Upon resettlement, cultural and language barriers may hinder access to formal mental health services, making refugees increasingly dependent on informal support networks, such as family and friends [5,15]. This disruption, coupled with reliance on informal networks, heightens the risk of mental health challenges, because refugees often lack the robust support systems they once had [14].

Social support has been identified as protective against psychological distress and suicidal ideation among refugees, with strong social networks promoting resilience and emotional well-being [2,15,39]. Psychosocial interventions that build social connectedness, reduce isolation, increase social capital, and improve access to mental health services have been shown to enhance refugees’ psychosocial well-being [7]. However, some studies suggested that not all social support interventions are equally effective. Vulnerable subgroups in refugee populations may require more tailored programs, because generalized interventions sometimes lead to a decline in mental health [7]. Weak or disrupted social networks have also been identified as a significant risk factor for suicide among refugees [14].

Research has indicated that whether social networks act as a risk or protective factor for refugees’ mental well-being may depend on the type of social capital involved—bonding or bridging [44]. Bonding social capital refers to relationships with family, friends, or other refugees who share the same ethnic background and plays a crucial role in preserving identity and providing a space to share collective experiences of displacement [45]. Individuals who have faced traumatic events are more likely to demonstrate resilience when they maintain or regain valued social identities [46]. Thus, intragroup interactions are a vital protective factor for mental health, offering a sense of belonging and emotional support during resettlement [44].

Rebuilding social networks in the resettlement context is essential for accessing critical resources such as employment, education, and housing. However, disrupted networks are not easily reestablished [47,48]. Bridging social capital, or relationships with members of the host society, can foster autonomy, confidence, and security, helping mitigate the negative effects of trauma and postmigration challenges on well-being [44]. For example, local residents can offer practical assistance with everyday challenges. Additionally, intergroup interactions can enhance refugees’ feelings of acceptance and belonging, reducing acculturative stress [49]. Therefore, intergroup contact can be an important protective factor for refugees’ mental health and overall well-being [44].

### Current study

The current study built on prior research that underscored the importance of social support and social networks for mental health among refugees. Recognizing the challenges refugees face—from disrupted social networks to inadequate culturally responsive care—the current research aimed to deepen our understanding of these dynamics in specific refugee communities. Using egocentric network data collected from 150 refugees in Arizona, this study adopted a community-based participatory research approach to gain insights from community members. Because Bhutan, Burma, Democratic Republic of Congo, Iraq, Somalia, and Syria rank among the top 10 nationalities of refugees resettled in Arizona [50], this study focused on these six subgroups.

The current pilot study aimed to address two key research questions: (a) How do sociodemographic characteristics vary across these six refugee subgroups? (b) How do social support, social network characteristics, and mental health—specifically, psychological distress and suicidal thoughts—differ across these subgroups? To our knowledge, this is the first study to employ egocentric network data to compare social networks and mental health indicators across Arizona’s diverse refugee subgroups. By illuminating these differences, we aim to inform culturally responsive mental health interventions to better serve the needs of these unique communities.

## Methods

### Participants

The current study used community-based participatory research, a collaborative approach that equitably involves community members, researchers, and other stakeholders in the research process and recognizes their unique strengths [51]. Community leaders from the six refugee groups were active partners in the study. The community leaders were involved through all phases of the study, creating a co-learning experience for them and the researchers. The community partners were equally involved in the conceptualization of the study, development and checking of the cultural appropriateness of the survey, back-translation of the survey, recruitment, survey administration, and dissemination of findings.

After receiving intensive training from the first author on research processes, the community partners recruited 150 refugee adults (25 from each group) using purposive and snowball sampling from July 3 to September 29, 2022. Inclusion criteria were: (a) being 18 years of age or older, (b) having migrated from Bhutan, Burma, Democratic Republic of Congo, Iraq, Somalia, or Syria to the United States through the U.S. Refugee Admissions Program (excluding immigrants or temporary migrants) and (c) currently residing in the Phoenix metropolitan area. Individuals were excluded if they were younger than 18 years of age, had migrated to the United States through non-refugee channels (e.g., family reunification, employment-based migration, or student visas), originated from countries other than the six specified above, or were not residing in the Phoenix metropolitan area.Our study employed a quantitative survey to examine patterns and trends among locally resettled refugee subgroups in Arizona, with the goal of informing culturally responsive and contextually grounded interventions. The geographic concentration of refugees in the state provided a unique opportunity to analyze how local resettlement systems, social networks, and service infrastructures influence refugee well-being. Focusing on a specific local context also enabled the detection of community-level patterns that may be obscured in broader national datasets, while contributing to a growing body of place-based integration research.

### Ethics statement and procedures

The institutional review board at Arizona State University approved all survey items and procedures on April 9, 2021 (STUDY00013753). To ensure linguistic and cultural validity, we utilized a team-based translation and back-translation process grounded in the CBPR framework [52]. A bilingual translator first produced the forward translation in Nepali, Burmese, Swahili, Arabic, and Somali, which was then reviewed by a diverse team of community leaders and bilingual researchers for conceptual and semantic equivalence. An independent translator then completed the back-translation. Any discrepancies were resolved collaboratively, with attention to cultural nuance and item clarity. Community partners assessed whether items were understandable, meaningful, and contextually appropriate. We then pilot-tested the final version of the translated surveys with a small sample (n = 5 per group) across the six groups [52]. Grounded in the CBPR approach, this participatory, iterative approach strengthened the content, face, and cultural validity of the translated survey and functioned as a key validation strategy in addition to the subsequent psychometric assessments.

Community partners administered the survey at each ethnic-based community organization for refugees or at participants’ homes. Both English and ethnic language versions of the survey were offered, allowing participants to select their preferred language. Although the survey was designed for self-administration, whenever a participant had literacy challenges, the community partner read the survey items to the participant and marked the participant’s responses. Surveys were administered only after obtaining written informed consent, which was presented on the second page of the survey. Each participant received a $30 Walmart gift card as compensation for their participation.

### Measures

#### Sociodemographic variables.

Sociodemographic characteristics of age (years), refugee group (1 = *Bhutanese*, 2 = *Burmese*, 3 = *Congolese*, 4 = *Iraqi*, 5 = *Somali*, 6 = *Syrian*), biological sex (1 = *male*, 2 = *female*), literate (1 = *yes*, 0 = *no*), marital status (1 = *married*, 0 = *not married*), employment status (1 = *employed*, 0 = *unemployed*), highest education level (1 = *never attended school or primary school*, 2 = *some high school or high school diploma*, 3 = *some college or college or advanced degree*), lived in a refugee camp (1 = *yes*, 0 = *no*), years lived in the United States, and English proficiency level were examined. English proficiency was measured by summing self-rated scores for speaking, listening, reading, and writing, each assessed on a 5-point Likert scale ranging from 1 (*very limited*) to 5 (*excellent*). Higher scores indicate greater overall proficiency.

#### Study variables.

Social support was measured by the 12-item Multidimensional Scale of Perceived Social Support [53]. Participants were asked how they felt about statements related to the source of social support, including family, friends, and significant other, with response options ranging on a 5-point Likert scale from 1 (*strongly disagree*) to 5 (*strongly agree*). Example items are “I get the emotional help and support I need from my family,” “There is a significant other with whom I can share my joys and sorrows,” and “My friends really try to help me.” An average score was calculated for all 12 items, with a higher score indicating a higher level of social support (Cronbach’s alpha = .95).

Social networks were assessed using each participant’s egocentric network data, in which each participant (ego) identified individuals in their social environment (alters) with whom they had meaningful interactions [54,55,56]. Participants were asked to name key social contacts and provide information about each alter (e.g., relationship type, contact frequency, perceived support), as well as the connections between those alters, if any. This methodology captures the structure and composition of each respondent’s immediate social network and has been employed in previous studies [54] to understand social support and resource access among refugee populations. During the self-administered survey, participants were asked about their significant social networks. “From time to time, most people discuss important matters with other people in person; by phone, text, or email; or through a social networking site such as Facebook, Instagram, etc. … Looking back over the past 6 months, who were the people with whom you discussed matters important to you?” The instructions and data collectors ensured that we did not collect any names of alters. Participants were then asked about their relationship with each alter. With this information, several network variables were measured: overall network structure, residence location of alters, relationship strength, relationship quality, interaction frequency, relationship type, relational homophily, and disclosure of suicidal thoughts.

Overall network structure was measured by network size, which is the number of alters nominated, and network density, which is ratio of actual ties to the total possible connections in the network. A higher network density indicates a denser network, meaning that more network actors know one another in a network. Residence location of alters was measured by the proportion living in the United States and the participant’s home country. For relationship strength, relationship length (years) was measured by asking, “How long have you known this person?” Emotional closeness to alters was measured with an item using a 5-point Likert scale that asked, “How emotionally close are you to this person?” Higher scores indicated more closeness. Average scores were calculated for both variables. For relationship quality, emotional support and informational support were measured using a 5-point Likert scale and the following questions: “How much emotional support do you receive from this person?” “How much informational support do you receive from this person?” Average scores were calculated for both variables, and higher scores indicated more support. Frequency of interaction was measured by a 5-point item: “How frequently do you have contact with this person?” Average scores were calculated, with higher scores indicating more frequent contact.

Relationship type was measured by asking, “What is this person’s relationship to you?” Multiple response options were available, and responses were categorized into four mutually exclusive relationship types: family, service providers (including refugee community organization staff member, social worker, and doctor), friends, and other (including neighbor and coworker). Proportions were calculated for each category. Relational homophily was assessed by whether the participant and alter shared the same properties with respect to age, sex, ethnicity, and refugee community. For the age variable, participants and alters were considered the same age if they were within 1 year of each other. All relational homophily variables were dichotomized.

Disclosure of suicidal thoughts was measured by past disclosure and future intent to disclose. Past disclosure was measured by asking, “If you had ever felt hopeless to continue living, did you tell this person?” Response options were yes or no. Future intent to disclose was measured by a 5-point Likert scale for the question, “In the future, if you were to feel hopeless to continue living, how likely would you tell this person?” Average scores were calculated, such that higher scores indicated a higher likelihood to disclose suicidal thoughts to an alter.

Psychological distress was assessed by the Refugee Health Screener-13, a culturally sensitive initial screening tool to identify psychological distress in refugees aged 14 or older [57]. This 13-item scale includes questions related to PTSD, depression, and anxiety symptoms, with response options ranging on a 5-point Likert scale from 0 (*not at all*) to 4 (*extremely*). A total score was calculated for all 13 items, with a higher score indicating a higher level of psychological distress (Cronbach’s alpha = .95).

Given that many refugees come from cultures with taboos against suicide [30], to be more culturally appropriate, the current study measured passive suicidal thoughts using the Wish to be Dead Scale [58]. This scale has been used with populations from cultures where suicide is stigmatized, including refugees, and has been shown to be an effective alternative approach to assessing suicide risk in a culturally responsive manner [59]. The original scale consists of 10 items such as “There have been times when I wished that I were dead” and “It occasionally crosses my mind that life is not worth living.” After discussions with community partners, we added an additional item, “If there is any way to end my life, I would use it,” to better capture the refugee communities’ passive suicidal thoughts. Response options ranged on a 6-point Likert scale from 1 (*strongly disagree*) to 6 (*strongly agree*). A total score was calculated for all 11 items, with a higher score indicating a higher level of suicidal thoughts (Cronbach’s alpha = .97).

### Data analysis

We first conducted descriptive statistical analyses of sociodemographic characteristics to gain a comprehensive overview of the sample. We also conducted descriptive analyses for the study variables: social support, social networks, and mental health. In addition, to compare sociodemographic characteristics by six refugee groups, chi-square tests and analysis of variance (ANOVA) were conducted. A post hoc power analysis using G*Power 3.1 indicated that, given α = .05, six groups, and a total sample size of *N* = 150 (n = 25 per group), the study had approximately 80% power to detect medium-to-large effects (f = 0.30) in Kruskal–Wallis group comparisons [60].

Before comparing social support, social networks, and mental health variables across six refugee groups, we first evaluated the assumptions for parametric analyses (i.e., ANOVA) by conducting normality tests. The Shapiro-Wilk test showed statistical significance for all study variables (*p* < .01), indicating that the variables were not normally distributed. Therefore, we used nonparametric tests for group comparisons. We conducted Kruskal–Wallis tests and post hoc pairwise Wilcoxon tests with a Bonferroni correction.

Missing data were minimal across variables, with the highest proportion being 2.0%. This low level of missingness was supported by the data collection procedures: during training, the first author emphasized to community partners the importance of reviewing each completed survey immediately and checking for missing responses before concluding the session, which helped ensure high data completeness. Descriptive statistics were computed using all available cases (pairwise deletion). For composite scales, scores were calculated using available-case (prorated) scoring, allowing participants with partial item responses to be retained. For the Kruskal–Wallis tests, listwise deletion was applied for each analysis, such that cases with missing data on the outcome variable were excluded from that specific test. Given that the proportion of missing data did not exceed levels generally considered unlikely to adversely affect study inferences (i.e., less than approximately 5.0% missingness), these procedures are unlikely to have introduced meaningful bias into the results [61]. All analyses were conducted using SPSS Version 28 [62]. Statistical significance was evaluated using a two-tailed alpha level of.05.

## Results

### Sociodemographic characteristics

The sample was evenly distributed across the six refugee subgroups, each making up 16.7%. The sample was 53.0% male and 47.0% female. Of the respondents, 32.9% had no literacy, 65.1% were married, and 44.7% were unemployed. Education levels varied: 31.3% had never attended school or only completed primary education, 45.3% had finished high school, and 23.3% had attended college or advanced education (see Table 1). In addition, 64.0% had lived in a refugee camp. Respondents’ average age was 41.4 years, average length of stay in the United States was 8.5 years, and average English proficiency score was 10.3.

**Table 1:** Sociodemographic Characteristics (*N* = 150).

Variable	*n* (%)
Refugee group	
Bhutanese	25 (16.7)
Burmese	25 (16.7)
Congolese	25 (16.7)
Iraqi	25 (16.7)
Somali	25 (16.7)
Syrian	25 (16.7)
Sex	
Male	79 (53.0)
Female	70 (47.0)
Literate	
No	49 (32.9)
Yes	100 (67.1)
Married	
No	52 (34.9)
Yes	97 (65.1)
Employment	
Unemployed	67 (44.7)
Employed	83 (55.3)
Education	
Never or primary school	47 (31.3)
High school	68 (45.3)
College or beyond	35 (23.3)
Lived in refugee camp	
No	54 (36.0)
Yes	96 (64.0)
Age	*M* = 41.42, *SD* = 14.74
Years in United States (range: 0–32 years)	*M* = 8.45, *SD* = 6.53
English proficiency (range: 2–20)	*M* = 10.28, *SD* = 5.59

*Note.* Sample size varied for each item based on missing values.

### Social support, social networks, and mental health variables

Table 2 presents descriptive statistics for social support, social networks, and mental health variables. The median score for social support was 4.33 (range = 1–5), indicating a high level of perceived social support. For social network variables, the median network size was 2.00 (range = 1–5), whereas network density was high at 1.00 (range = 0–1). The median proportion of network members living in the U.S. was 100.0%. The median duration of relationships was 18.00 years and the median emotional closeness was 4.40 (range = 1.4–5.0). In terms of relationship quality, the median scores for emotional support and informational support were 4.50 and 4.33, respectively (range = 1–5).

**Table 2:** Descriptive Statistics of Social Support, Social Networks, and Mental Health Variables (*N* = 150).

Variable	Mean	Median	Range
Social support	4.16	4.33	1–5
Social networks			
Overall network structure			
Network size	2.53	2.00	1–5
Network density	0.85	1.00	0–1
Residence location of alter			
United States	0.87	1.00	0–1
Home country	0.08	0.00	0–1
Relationship strength			
Relationship duration (years)	18.08	18.00	0–50
Emotional closeness	4.29	4.40	1.4–5.0
Relationship quality			
Emotional support	4.33	4.50	1–5
Informational support	4.25	4.33	1–5
Contact frequency	4.33	4.50	1–5
Relationship type			
Family	0.67	0.80	0–1
Service provider	0.04	0.00	0–1
Friend	0.26	0.00	0–1
Other	0.02	0.00	0–0.33
Relational homophily			
Same age	0.15	0.00	0–1
Same sex	0.47	0.50	0–1
Same ethnicity	0.92	1.00	0–1
Same refugee community	0.86	1.00	0–1
Disclosure of suicidal thoughts			
Past disclosure	0.32	0.00	0–1
Future intent to disclose	4.25	4.60	0.67–5.00
Psychological distress	9.97	5.00	0–50
Suicidal thoughts	14.77	11.00	11–66

The median frequency of interaction was high, with a score of 4.50 (range = 1–5). The median proportions of relationship types classified as family, service providers, friends, and others were 80.0%, 0.0%, 0.0%, and 0.0%, respectively. Relational homophily showed that the median proportions of network members of the same age, sex, ethnicity, and refugee community were 0.0%, 50.0%, 100.0%, and 100.0%, respectively. For disclosure of suicidal thoughts to network members, the median proportion of past disclosure was 0.0% and the median future intent to disclose score was 4.60. Psychological distress had a median score of 5.00 (range = 0–50) and suicidal thoughts had a median score of 11.00 (range = 11–66).

### Sociodemographic characteristics by refugee groups

Chi-square and ANOVA tests revealed significant differences in sociodemographic characteristics across groups (Table 3). The proportion of male participants varied significantly across six refugee groups (χ^2^ = 17.17, *p* < .01), with the highest rate among Somali refugees (84.0%) and the lowest among Burmese refugees (28.0%). Literacy rates also differed significantly (χ^2^ = 23.71, *p* < .001), with Bhutanese refugees having the highest rate (96.0%) and Burmese refugees having the lowest rate (40.0%). The proportion of married respondents differed significantly (χ^2^ = 15.73, *p* < .01), with the highest level among Bhutanese refugees (84.0%) and lowest among Congolese refugees (45.8%). Employment rates varied significantly (χ^2^ = 22.15, *p* < .001), with Bhutanese refugees having the highest rate (80.0%) and Syrian refugees having the lowest rate (28.0%). Educational levels also differed significantly (χ^2^ = 31.33, *p* < .001); Bhutanese refugees had the highest proportion with only primary education (56.0%) and Iraqi refugees had the highest proportion with college or advanced education (56.0%). The experience of living in a refugee camp was significantly different among groups (χ^2^ = 94.10, *p* < .001), with all Bhutanese refugees but no Iraqi refugees having lived in a camp. Average age differed significantly (*F* = 3.28, *p* < .01), with Somali refugees being the oldest (49.7 years) and Burmese participants being the youngest (35.8 years). Years in the United States also varied significantly (*F* = 53.90, *p* < .001), with Somali refugees having the longest average stay (17.21 years) and Syrian refugees having the shortest stay (1.28 years). English proficiency scores differed significantly (*F* = 5.11, *p* < .001), with Iraqi refugees scoring the highest (13.72) and Syrian refugees scoring the lowest (7.04).

**Table 3:** Sociodemographic Characteristics by Refugee Group.

Variable	Bhutanese	Burmese	Congolese	Iraqi	Somali	Syrian	
	(*n* = 25)	(*n* = 25)	(*n* = 25)	(*n* = 25)	(*n* = 25)	(*n* = 25)	
	*n* (%) or *M* (*SD*)	*n* (%) or *M* (*SD*)	*n* (%) or *M* (*SD*)	*n* (%) or *M* (*SD*)	*n* (%) or *M* (*SD*)	*n* (%) or *M* (*SD*)	χ^2^ or *F*
Sex							17.17*
Male	15 (60.0)	7 (28.0)	13 (52.0)	11 (45.8)	21 (84.0)	12 (48.0)	
Female	10 (40.0)	18 (72.0)	12 (48.0)	13 (54.2)	4 (16.0)	13 (52.0)	
Literate							23.71**
No	1 (4.0)	15 (60.0)	12 (48.0)	4 (16.0)	9 (36.0)	8 (33.3)	
Yes	24 (96.0)	10 (40.0)	13 (52.0)	21 (84.0)	16 (64.0)	16 (66.7)	
Married							15.73*
No	4 (16.0)	5 (20.0)	13 (54.2)	13 (52.0)	11 (44.0)	6 (24.0)	
Yes	21 (84.0)	20 (80.0)	11 (45.8)	12 (48.0)	14 (56.0)	19 (76.0)	
Employment							22.15**
Unemployed	5 (20.0)	10 (40.0)	16 (64.0)	12 (48.0)	6 (24.0)	18 (72.0)	
Employed	20 (80.0)	15 (60.0)	9 (36.0)	13 (52.0)	19 (76.0)	7 (28.0)	
Education							31.33**
Never or primary	14 (56.0)	5 (20.0)	8 (32.0)	2 (8.0)	7 (28.0)	11 (44.0)	
High school	9 (36.0)	14 (56.0)	14 (56.0)	9 (36.0)	14 (56.0)	8 (32.0)	
College or beyond	2 (8.0)	6 (24.0)	3 (12.0)	14 (56.0)	4 (16.0)	6 (24.0)	
Lived in refugee camp							94.10**
No	0 (0.0)	5 (20.0)	4 (16.0)	25 (100.0)	1 (4.0)	19 (76.0)	
Yes	25 (100.0)	20 (80.0)	21 (84.0)	0 (0.0)	24 (96.0)	6 (24.0)	
Age	43.88 (14.23)	35.80 (9.68)	43.00 (20.77)	37.52 (13.38)	49.72 (14.26)	38.60 (10.02)	3.28*
Years in United States	10.16 (2.01)	7.58 (3.88)	3.29 (2.71)	11.42 (2.52)	17.21 (7.33)	1.28 (2.23)	53.90**
English proficiency	10.12 (5.23)	11.08 (5.16)	8.32 (4.84)	13.72 (5.65)	11.40 (5.55)	7.04 (4.97)	5.11**

*p < .01. **p < .001

### Social support, social networks, and mental health status by refugee group

Kruskal-Wallis H tests (hereafter, H) revealed significant differences in social support, social networks, and mental health status across the six refugee groups (Table 4).

**Table 4:** Social Support, Social Networks, and Mental Health Variables by Refugee Group.

Variable	Bhutanese (a)	Burmese (b)	Congolese (c)	Iraqi (d)	Somali (e)	Syrian (f)	Kruskal-Wallis H	Bonferroni-adjusted Wilcoxon p-values
	(*n* = 25)	(*n* = 25)	(*n* = 25)	(*n* = 25)	(*n* = 25)	(*n* = 25)		
	*M* rank	*M* rank	*M* rank	*M* rank	*M* rank	*M* rank		
Social support	84.24	96.96	53.88	78.72	86.42	52.78	22.74***	c < b, p_adj = .010 f < b, p_adj = .005
Social networks								
Overall network structure								
Network size	128.76	95.52	60.04	74.42	50.26	44.00	72.64***	b–f < a, p_adj < .001 e, f < b, p_adj < .001 f < d, p_adj = .014
Network density	72.00	51.36	61.22	53.80	63.73	37.38	16.96**	b < a, p_adj = .018 d < a, p_adj = .037 f < a, p_adj < .001
Residence location								
Living in United States	89.62	61.44	58.84	83.06	74.00	86.04	21.33***	b < a, p_adj = .012 c < a, p_adj = .025
Living in home country	66.00	94.76	79.12	71.92	75.20	66.00	22.92***	a < b, p_adj = .008 f < b, p_adj = .008
Relationship strength								
Relationship duration (years)	87.00	80.16	62.54	53.98	100.44	68.88	19.22**	d < a, p_adj = .024 d < e, p_adj = .003
Emotional closeness	77.04	57.58	62.44	73.22	96.52	83.54	14.34*	b < e, p_adj = .014
Relationship quality								
Emotional support	76.22	55.24	67.22	74.24	93.08	84.38	12.49*	b < e, p_adj = .014
Informational support	75.38	62.30	58.06	73.30	95.48	85.92	13.86*	c < e, p_adj = .034
Contact frequency	82.16	32.86	70.64	76.26	95.58	93.23	37.73***	b < adef, p_adj < .001
Relationship type								
Family	87.58	67.50	70.56	49.28	91.08	87.00	19.37**	d < a, p_adj = .004 d < e, p_adj = .016
Service providers	79.94	74.66	74.24	77.78	77.88	68.50	4.26	N.S.
Friends	61.03	81.82	83.56	98.02	57.18	69.38	18.71**	a < d, p_adj = .017 e < d, p_adj = .007
Other	78.78	85.08	70.00	73.18	70.00	75.96	.07	
Relational homophily								
Same age	84.72	83.98	74.24	77.66	72.80	59.60	8.31	N.S.
Same sex	72.62	86.44	87.54	89.06	52.74	64.60	14.26*	N.S.
Same ethnicity	83.30	81.56	75.14	59.74	80.90	71.86	13.37*	N.S.
Same refugee community	90.44	48.08	79.42	61.12	91.34	82.60	33.40***	b < a, p_adj < .001 b < e, p_adj = .001 b < f, p_adj = .028 d < a, p_adj = .029 d < e, p_adj = .028
Disclosure of suicidal thoughts								
Past disclosure	73.30	74.90	96.64	50.36	50.60	107.20	47.27***	a < f, p_adj = .016 d, e < c, f, p_adj < .001
Future intent to disclose	95.52	54.50	48.50	85.18	90.96	75.35	27.55***	b < a, p_adj = .001 c < a, p_adj = .003 c < d, p_adj = .032 c < e, p_adj = .032 b < e, p_adj = .028
Psychological distress	72.82	48.96	104.30	67.84	52.68	106.40	41.13***	c > a, p_adj = .040 c > b, p_adj < .001 c > d, p_adj = .003 c > e, p_adj = .003 f > b, p_adj < .001 f > d, p_adj = .007 f > e, p_adj = .003
Suicidal thoughts	86.70	62.16	81.80	68.12	62.14	92.08	18.95**	b < f, p_adj = .045

*Note.* Only pairwise Wilcoxon comparisons that remained statistically significant after Bonferroni correction are presented. Reported p-values are Bonferroni-adjusted (p_adj). Non-significant comparisons are omitted for clarity.

N.S. = not significant, *p <.05. **p <.01. ***p <.001.

#### Social support.

We found significant differences in social support among the six refugee groups (H = 22.74, *p* < .001). The highest mean rank for social support was observed among Burmese refugees (96.96), in contrast to Syrian refugees, who had the lowest mean rank (52.78). Post hoc tests showed that Congolese and Syrian refugees had significantly lower social support than Burmese refugees.

#### Social networks.

For overall network structure, results showed significant differences among six refugee groups, including network size (H = 72.64, *p* < .001) and network density (H = 16.96, *p* < .01). Specifically, post hoc testing on network size indicated that Syrian and Somali refugees had significantly smaller network sizes than Burmese and Iraqi refugees, who in turn had significantly smaller networks than Bhutanese refugees. Bhutanese refugees reported the largest networks (mean rank = 128.76), whereas Syrian and Somali refugees had the smallest networks (mean ranks = 44.00 and 50.26, respectively). Regarding network density, post hoc testing showed that Syrian, Burmese, and Iraqi refugees had significantly lower network density than Bhutanese refugees. Network density was highest among Bhutanese refugees (mean rank = 72.00) and lowest among Syrian (mean rank = 37.38), Burmese (mean rank = 51.35), and Iraqi refugees (mean rank = 53.80).

Regarding the residence location of network members, significant group differences were observed (H = 21.33, *p* < .001). Post hoc tests further indicated that Congolese and Burmese refugees had significantly lower proportions of U.S.-based network members than Bhutanese refugees. Mean ranks of these networks were highest among Bhutanese refugees (89.62) and lowest among Congolese (58.84) and Burmese refugees (61.44). Conversely, Burmese refugees had the highest proportion of network members living in their home country (mean rank = 94.76), whereas Bhutanese and Syrian refugees had the lowest (mean rank = 66.00), with a significant difference across refugee groups (H = 22.92, *p* < .001). Post hoc testing confirmed that these groups differed significantly from one another.

Regarding relationship strength, we found significant differences in relationship duration (H = 19.22, *p* < .01) and emotional closeness (H = 14.34, *p* < .05). Post hoc tests for relationship duration indicated that Iraqi refugees reported significantly shorter relationships than Somali and Bhutanese refugees, and post hoc results for emotional closeness showed that Burmese refugees reported significantly lower emotional closeness than Somali refugees. The average relationship duration was longest among Somali (mean rank = 100.44) and Bhutanese refugees (mean rank = 87.00), and shortest among Iraqi refugees (mean rank = 53.98). Average emotional closeness was highest among Somali refugees (mean rank = 96.52) and lowest among Burmese refugees (mean rank = 57.58).

Furthermore, we found significant differences in relationship quality in terms of emotional support (H = 12.49, *p* < .05) and informational support (H = 13.86, *p* < .05) among refugee groups. Post hoc tests showed that Burmese refugees reported significantly lower emotional closeness than Somali refugees, and that Congolese refugees reported significantly lower emotional support than Somali refugees. Average emotional support was highest among Somali refugees (mean rank = 93.08) and lowest among Burmese refugees (mean rank = 55.24). Average informational support was highest among Somali refugees (mean rank = 95.48) and lowest among Congolese refugees (mean rank = 58.06). Average contact frequency significantly differed across groups (H = 37.73, *p* < .001). Post hoc comparisons revealed that Burmese refugees had significantly lower contact frequency than Syrian, Somali, Bhutanese, and Iraqi refugees. Mean ranks showed that contact frequency was highest among Syrian (93.23), Somali (95.58), Bhutanese (82.16), and Iraqi refugees (76.26) and lowest among Burmese refugees (32.86).

Regarding relationship types, significant group differences were found for both family networks (H = 19.37, *p* < .01) and friend networks (H = 18.71, *p* < .01). Post hoc tests indicated that Iraqi refugees had significantly fewer family networks than Bhutanese and Somali refugees. Mean ranks showed that family networks were most prevalent among Somali refugees (91.08) and Bhutanese refugees (87.58) and least prevalent among Iraqi refugees (mean rank = 49.28). For friend networks, post hoc comparisons further showed that Bhutanese and Somali refugees had significantly lower proportions of friend networks than Iraqi refugees. Correspondingly, mean rank indicated that friend networks were most prevalent among Iraqi refugees (98.02) and least prevalent among Somali (57.18) and Bhutanese refugees (61.3).

Relational homophily significantly differed among the six groups in the proportion of same-sex network members (H = 14.26, *p* < .01). The proportion was highest among Iraqi refugees (mean rank = 89.06) and lowest among Somali refugees (mean rank = 52.74). The proportion of same-ethnicity network members also significantly differed (H = 13.37, *p* < .05)—highest among Bhutanese refugees (mean rank = 83.30) and lowest among Iraqi refugees (mean rank = 59.74). However, post hoc tests did not reveal significant group differences for either same sex or same ethnicity networks. The proportion of networks from the same refugee community was significantly different (H = 33.40, *p* < .001). Post hoc tests showed that Burmese and Iraqi refugees had significantly lower proportions of same refugee community network members than Bhutanese and Somali refugees, while Burmese refugees also had significantly lower proportions than Syrian refugees. Mean ranks indicated that same-community networks were most prevalent among Somali (91.34) and Bhutanese refugees (90.44) and least prevalent among Burmese refugees (48.08).

#### Mental Health.

We also found significant group differences in both past disclosure of suicidal thoughts (H = 47.27, *p* < .001) and future intent to disclose (H = 27.55, *p* < .001). Post hoc comparisons for past disclosure indicated that Bhutanese refugees reported significantly lower past disclosure than Syrian refugees. In addition, Iraqi and Somali refugees reported significantly lower disclosure than Congolese and Syrian refugees. Mean ranks showed that past disclosure was highest among Syrian refugees (107.20) and lowest among Iraqi refugees (50.36). For future intent to disclose, post hoc tests showed that Burmese and Congolese refugees had significantly lower intent to disclose than Bhutanese refugees, and Congolese refugees also reported lower intent than Iraqi and Somali refugees. Correspondingly, mean ranks indicated that future disclosure intent was highest among Somali (90.96) and Bhutanese refugees (95.52) and lowest among Congolese (48.50) and Burmese refugees (54.50).

Results reveal significant differences in psychological distress (H = 41.13, *p* < .001) and suicidal thoughts (H = 18.95, *p* < .01) among the six refugee groups. For psychological distress, post hoc comparisons indicated that Bhutanese, Burmese, Iraqi, and Somali refugees had significantly lower levels of distress than Congolese and Syrian refugees. Mean ranks showed that psychological distress was highest among Congolese (104.30) and Syrian refugees (106.40) and lowest among Burmese refugees (48.96).

For suicidal thoughts, post hoc tests showed that Burmese refugees had significantly lower levels of suicidal thoughts than Syrian refugees. Correspondingly, mean ranks indicated that suicidal thoughts were highest among Syrian refugees (92.08) and lowest among Somali (62.14) and Burmese (62.16) refugees.

## Discussion

The current pilot study aimed to provide a descriptive overview of sociodemographic characteristics, social support, social network structure, and mental health status (psychological distress and suicidal thoughts) among Bhutanese, Burmese, Congolese, Iraqi, Somali, and Syrian refugees in Arizona. The findings highlight some similar patterns, such as the shared dependence on interpersonal relationships, common concerns about mental health communication and the salience of cultural identity in building social ties among refugee subgroups. There were also substantial variations in these characteristics across refugee subgroups. These findings emphasize the need for culturally responsive, adaptable, scalable, nuanced approaches to mental health by leveraging social support assets across refugee subgroups.

Further, by examining these attributes in each group, we gained insights into the potential influences of demographic factors and social networks on mental health outcomes, though causal interpretations remain limited by the descriptive nature of our study. Specifically, the Bhutanese refugee group in our study exhibited high literacy, which contrasts with previous research findings, and high employment rates, which aligns with prior research [32]. These refugees had the most extensive same-ethnicity networks in the United States. Although these close-knit, culturally familiar networks likely provide a strong source of emotional support, the high perceived likelihood of future disclosure of suicidal thoughts suggests possible underlying mental health needs in this group. The tendency of Bhutanese refugees to build relationships primarily in their own communities can both support and limit their integration experiences. Although relational homophily may foster comfort and shared understanding, it may also restrict access to diverse perspectives and resources. These findings align with previous studies discussing the “cocooning effect,” by which heavy reliance on co-ethnic networks can limit long-term mobility and integration into broader society [63,64]. This highlights an important area for further investigation, such as examining how network density and ethnic homogeneity may affect mental health among Bhutanese refugees. Future studies could explore whether tight-knit networks offer sufficient mental health support or limit access to broader resources that might enhance psychological well-being.

Burmese refugees in our study showed unique social and mental health characteristics. They reported the lowest literacy rate, consistent with prior research [15]. Although these participants had the highest levels of social support, they showed low interaction frequency and emotional closeness with network members, most of whom resided in their home country. Their low levels of psychological distress suggest that social support may serve as a protective factor, aligning with earlier findings [15]. Maintaining distant social ties may provide sufficient emotional stability, potentially due to the cultural and familial familiarity embedded in these relationships. However, limited interaction frequency and physical distance from network members could pose challenges for long-term social integration in the United States [65]. Mental health interventions for Burmese refugees may benefit from strategies that support the development of local networks while preserving meaningful connections abroad, helping them balance cultural continuity with the demands of adapting to a new environment [16].

In our study, Congolese refugees had limited U.S.-based network support and low levels of informational support, which could indicate a degree of social isolation. Additionally, with the fewest married participants and a lower likelihood of disclosing suicidal thoughts, Congolese refugees may lack trusted support systems. These findings are somewhat consistent with previous research, which found that a low sense of connectedness and poor interpersonal relationships are linked to higher levels of suicidal ideation among Congolese refugees [5]. Although we could not determine the effects of these characteristics on mental health, the descriptive data suggest that programs aimed at enhancing informational support and creating safe spaces for mental health conversations could be beneficial for this group.

Iraqi refugees in our study were characterized by high levels of education, strong English proficiency, and predominantly friend-based networks, with relatively few family ties. The low rate of suicidal thought disclosure in this group suggests a positive adjustment process. Although descriptive patterns suggested that Iraqi refugees had fewer same-ethnicity connections and shorter relationship durations, post hoc tests showed that same-ethnicity network proportions did not differ significantly across refugee groups. Thus, the social networks of Iraqi refugees appear shaped more by educational and linguistic factors than by ethnic homophily. These findings point to the potential role of language proficiency and friend-oriented networks in supporting mental health and adaptation among Iraqi refugees. Programs that foster both community belonging and opportunities for cross-cultural social engagement may be especially beneficial for this group [16,44].

Somali refugees, primarily male and the oldest group in our study, reported high interaction frequency, emotional closeness, and family-centered networks. Given their longer residency in the United States and well-established networks, they had lower levels of suicidal thoughts, suggesting that family-based support networks might play a critical role in their mental health. Although descriptive, these findings are consistent with previous research emphasizing family support as a protective factor [16]. Programs that support family-centered networks could be particularly beneficial for Somali refugees, providing continuity in their support systems.

Syrian refugees exhibited distinct challenges, with the lowest employment and English proficiency rates, limited U.S. residency, and small, dispersed networks. Their high levels of psychological distress and suicidal thoughts suggest that restricted social integration and limited economic opportunities may exacerbate mental health vulnerabilities. The elevated rate of past disclosure of suicidal thoughts suggests some openness yet also emphasizes the urgent need to address mental health needs. Although descriptive, these findings highlight the potential necessity for targeted social support and mental health resources for Syrian refugees, particularly those focused on fostering local connections, improving language skills, and promoting economic stability to reduce isolation and alleviate distress [40,41].

Overall, our findings highlight both shared trends and illuminate group-specific disaggregated challenges. These insights support feasible, community-informed strategies that can be embedded into mental health support systems within existing social infrastructures and refugee networks, which can be scaled across refugee subgroups.

Additionally, findings from disaggregated refugee subgroup data highlight important similarities and differences in mental health needs and social network assets. These nuanced insights underscore the need to move beyond a one-size-fits-all approach, as mental health needs, social support, and social network characteristics uniquely interact within each group. Although the feasibility of individualized tailoring for each nationality or cultural group might be challenging and not practical due to resource constraints, leveraging community-level social network data can offer scalable, culturally responsive solutions. Identifying and mobilizing community assets, such as ethnic peer networks, faith-based leaders, and trusted service providers, holds promise for effectively addressing diverse mental health needs locally and nationally.

Prior research supports integrating social networks into refugee mental health interventions demonstrating enhanced psychological wellness and social connectedness [66,67]. Effective community-level mental health strategies include peer-led models [67], community gardening programs [68,69], mental health first aid training [70], and skill-based group interventions such as financial literacy workshops [71,72]. Such strategies align well with collectivist cultural norms, fostering shared resilience and autonomy [73,74]. Additionally, healing-centered approaches that emphasize community strengths, resilience, identity, collective cultural healing, and future aspirations have demonstrated effectiveness in promoting mental well-being [75]. Leveraging existing cultural and community networks to create inclusive and safe spaces can significantly enhance refugees’ overall psychological health and social integration [75].

Thus, a social network approach is urgently needed across research, policy, and practice to address refugee mental health both at the group and sub-group levels. Mental health practitioners and policymakers should consider the varying social support, network composition, psychological distress, and suicidal thoughts experienced by each group and prioritize the development of culturally responsive mental health interventions that leverage social support networks among refugees [76]. Additionally, strengthening local partnerships with ethnic community-based organizations can expand vital support networks while fostering social cohesion and promoting resilience across refugee communities [6,76]. These efforts can also facilitate cross-cultural engagement, which is critical for refugees’ long-term integration and well-being. Additionally, given the sensitive nature of suicide disclosures, it is vital to increase awareness and provide training for community leaders and service providers on how to address mental health concerns in culturally responsive ways. This would empower refugees to seek help more openly and reduce stigma surrounding mental health issues [16]. Researchers should investigate how these social support dynamics evolve over time, particularly as refugees settle into their new environments, to better understand the relationships of social support, social networks, and mental health outcomes. This knowledge will be crucial for developing effective, long-term support mechanisms that enhance well-being and resilience across diverse refugee communities.

## Limitations

Our study had several limitations. First, the cross-sectional design limited our ability to infer causal relationships between variables. Researchers should gather longitudinal data to better assess causal links among sociodemographic characteristics, social support, social networks, and mental health status among refugees. Second, the use of nonprobability sampling and a small sample restricts generalizability to all refugees in Arizona. Scholars could improve representativeness by employing random sampling with a broader sampling frame. Further, findings from a quantitative study from a localized sample may not be generalizable in a conventional sense. Future studies could use a mixed methodology to generate qualitative findings to further enrich the quantitative findings. Third, reliance on egocentric network data, which capture only immediate social environments, may not fully represent broader social networks—particularly in cases where structural factors influence outcomes. This approach may overlook indirect influences and important dynamics, such as bridging ties and network clusters, that are essential for understanding social cohesion and resilience. Despite these limitations, our study offers the first detailed insights into the characteristics of personal networks among refugee subgroups in Arizona and how these networks vary across groups. Given the scarcity of social network studies focused on refugees, we hope our work inspires further research in this area. Researchers should consider incorporating sociocentric network analysis to provide a more comprehensive understanding of refugee networks. Combining egocentric and sociocentric data would enable examination of both individual and community-level connections, leading to more robust and generalizable findings.

## Conclusion

The current study highlights the significant differences in sociodemographic characteristics, social support, social network characteristics, and mental health across six refugee subgroups in Arizona. Our study findings highlight both shared trends and group-specific barriers. These broader findings support scalable, culturally responsive strategies that leverage existing social infrastructure that can be embedded into community refugee networks. These findings also emphasize the need to promote cross-cultural engagement, expand community support, and foster diverse networks. Such strategies are essential for enhancing social integration and mental health outcomes, ultimately contributing to the creation of inclusive, resilient communities. Addressing these dimensions is critical for supporting refugee well-being and facilitating their long-term integration into society.
